# An oral delivery vehicle based on konjac glucomannan acetate targeting the colon for inflammatory bowel disease therapy

**DOI:** 10.3389/fbioe.2022.1025155

**Published:** 2022-11-10

**Authors:** Chuang Wang, Zhenzhao Guo, Jialuo Liang, Na Li, Rijian Song, Lei Luo, Yilong Ai, Xia Li, Shunqing Tang

**Affiliations:** ^1^ Foshan Stomatology Hospital and School of Medicine, Foshan University, Foshan, China; ^2^ Biomedical Engineering Institute, Jinan University, Guangzhou, China; ^3^ Guangdong Engineering Research Center of Oral Restoration and Reconstruction, Affiliated Stomatology Hospital of Guangzhou Medical University, Guangzhou, China

**Keywords:** konjac glucomannan, nanoparticle, colonic macrophages, targeting delivery, inflammatory bowel disease

## Abstract

Orally administered colon-targeted delivery vehicles are of major importance in the treatment of inflammatory bowel disease (IBD). However, it remains a challenge to maintain the integrity of such delivery vehicles during treatment, particularly in the gastric environment, which may cause untimely drug release before reaching the targeted colon. Herein, an oral colon-targeted drug delivery system (OCDDS) based on acetylated konjac glucomannan (AceKGM) has been developed in this work, which accomplishes colonic localization release and targets local inflammatory macrophages. The AceKGM nanoparticle-loading curcumin (Cur) was successfully fabricated by emulsion solvent evaporation techniques. DLS, AFM, and SEM were used in order to evaluate the nanoparticles’ diameter as well as their *in vitro* drug release profile, and reactive oxygen species (ROS) scavenging results showed that the OCDDS considerably retained the activity of Cur treated with simulated gastric fluid (SGF) and controllably released in simulated intestinal fluid (SIF). In addition, the adhesion experiment results indicated that the nanoparticle could accumulate on the colonic macrophages. Evaluations in colitis mice showed that the treatment significantly alleviated the symptoms of colitis by decreasing the local level of myeloperoxidase (MPO) and the disease activity index (DAI) score in mice. In summary, the results of our research demonstrate that Cur–AceKGM nanoparticles exhibit significantly improved therapeutic efficacy compared to orally administered free Cur and can be developed as an effective drug delivery vehicle for IBD treatment.

## Graphical Abstract

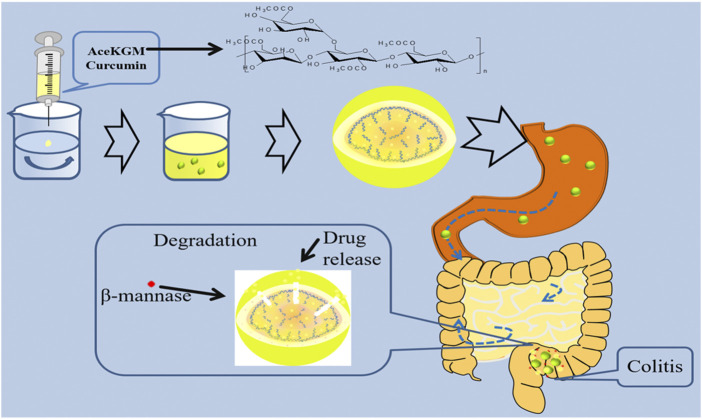

## Highlights

AceKGM nanoparticles were degraded and released the loading drug in the colon.
AceKGM nanoparticles targeted colonic macrophages.
AceKGM nanoparticles loading curcumin had therapeutic efficacy against IBD.

## 1 Introduction

Inflammatory bowel disease (IBD) is a series of chronic disorders characterized by destruction and recurrent tissue disorders caused by dysregulated immune responses (Abraham and Cho, 2009). In recent decades, IBD has been designated as a contemporary refractory disease by the World Health Organization (WHO) due to the rise in morbidity (Ng et al., 2017). When it comes to the management of disorders that affect the colon, colon-targeted formulations of medications that can be taken orally are of critical importance. However, oral drugs frequently encounter a series of extreme conditions such as pH changes, digestive enzymes, and physical squeezes before reaching the targeted colon. Moreover, the drugs are rapidly eliminated by diarrhea, making the management of IBD a serious medical challenge.

To address these problems, a delivery vehicle capable of delivering the drug exclusively to the target site would be a desirable alternative. Multiple studies have proven that nanoparticle-based drug delivery vehicles are a potential and encouraging option for targeted IBD therapy (Lu et al., 2016; Louiselle et al., 2020; Zhao et al., 2021). Importantly, the nanoparticle has a large capacity for drug loading. Furthermore, recent studies have demonstrated that nanoparticles can accumulate to a greater extent in the inflamed intestinal mucosa and prolong the resident duration at the site of inflammation because they can be taken up by the immune cells in IBD to a significant level (Lamprecht et al., 2001; Ali et al., 2014). Hence, by degrading the nanoparticles, the encapsulated drug is then released at the desired site. This targeted approach has been effectively applied in the specific delivery of various drugs for the treatment of IBD.

Developing a delivery vehicle necessitates a well-designed functioning material. Nanoparticles based on natural polysaccharides such as chitosan, konjac glucomannan (KGM), and xanthan gum have attracted considerable attention in drug delivery research because of their physicochemical and biological advantages (Tang et al., 2018; Sethi et al., 2020; Hu and Luo, 2021). Among these polysaccharides, KGM is a promising candidate due to its desirable features such as biocompatibility, biodegradability, low cost, and bioactivities. In the past several decades, KGM and its derivatives have played an important role in the development of colon-targeted delivery vehicles (Xu et al., 2013; Zhang et al., 2014; Huang et al., 2015; Behera and Ray, 2016). KGM is regarded as an indigestible dietary fiber since it is not digested by digestive enzymes in the human upper gastrointestinal tract. However, KGM can be specifically degraded by colon β-mannanase present in the colon. Nevertheless, the majority of research has focused on the swelling and biodegradability of KGM delivery vehicles. Importantly, due to complications such as diarrhea during the colon inflammatory stage, a challenge in this domain is the controlled and sustained release and improving the bioavailability of drugs. Recently, our group revealed that acetyl plays a crucial role in promoting macrophage targeting of KGM; AceKGM can specifically bind to mannose receptors which are abundantly expressed on macrophages (Wang et al., 2020). This binding selectivity enables AceKGM nanoparticles to target colonic macrophages and enhance medication absorption.

Curcumin (Cur), a natural polyphenol isolated from the rhizomes of turmeric, possesses potential pharmacological effects, including anti-inflammatory, antioxidant, antineoplastic, immunomodulatory, and anticarcinogenic activities. It has been extensively used in IBD treatment due to its ability to efficiently downregulate inflammatory cytokines, eliminate free radicals, and promote mucosal healing (Song et al., 2010; Baliga et al., 2012). Moreover, clinical research studies have demonstrated that Cur is pharmacologically safe for humans even at high doses (Gupta et al., 2013). Unfortunately, due to its low solubility in aqueous mediums and instability at neutral and basic pH conditions, Cur has limited medicinal potential, resulting in minimal bioavailability (Arya and Pathak, 2014). Consequently, there is a growing emphasis on the development of an optimal mechanism for delivering Cur to colitis tissue, such as pellets, micelles, and nanoparticles (Lamprecht, 2010; Zhang and Merlin, 2018).

Considering the necessity of developing an oral colon-targeted delivery vehicle, we encapsulated Cur in AceKGM nanoparticles for targeted therapy of colitis. The Cur-loaded AceKGM nanoparticles (Cur–AceKGM NPs) were created using emulsion solvent evaporation. In addition, the size, drug-loading capacity, and structure of Cur–AceKGM NPs were characterized by SEM, AFM, ATR-FTIR, and X-ray, and the drug release profiles and activity were investigated in SGF and SIF. Then, the biocompatibility and cell adhesion of nanoparticles were also evaluated. The therapeutic efficacy of the Cur–AceKGM NPs was evaluated using an *in vivo* mouse IBD model. The study not only benefits the application of natural polysaccharides but also provides a novel strategy for developing an oral colon-targeted delivery vehicle, especially for delivering hydrophobic drugs.

## 2 Materials and methods

### 2.1 Materials

KGM (Mn = 6.9 × 10^5^ Da) was purchased from Chengdu Root Industry Co., Ltd. (Chengdu, China). Dextran sulfate sodium (DSS), Cur, dexamethasone, lipopolysaccharide (LPS), interleukin-4 (IL-4), β-mannase, and pepsase were purchased from Sigma-Aldrich. Trifluoroacetic anhydride (TFAA), 1,8-diazabicyclo (5,4,0)-7-undecene (DBU), polyethylene glycol succinate (TPGS), and 1,1,1,3,3,3-hexafluoro-2-isopropanol (HFIP) were purchased from Shanghai Aladdin Bio-Chem Technology Co., Ltd. (Shanghai, China). Dulbecco’s modified Eagle’s medium (DMEM) and fetal bovine serum (FBS) were purchased from Gibco (Atlanta, GA). The Cell Counting Kit-8 (CCK-8) was purchased from Beyotime Biotech (Jiangsu, China). SGF was prepared by adding 0.1 N HCl, 2 g NaCl, and 3.5 g pepsase to 1 L deionized water. SIF was prepared by adding 102 mg KH_2_PO_4_ and 155 mg Na_2_HPO_4_·2H_2_O to 1 L SGF; subsequently, 0.01 M NaOH solution was used to adjust pH to 7.4, and then 4 mg β-mannase was added. Unless stated otherwise, the other chemicals were of analytical grade and were used without further treatment.

### 2.2 Preparation of Cur-loaded AceKGM nanoparticles

AceKGM was prepared according to the preparation procedure in our previous study (Wang et al., 2020). The degree of substitution of the obtained AceKGM was 3.0, according to the acid–base titration calculations. Nanoparticles were prepared by an emulsion solvent evaporation method. The major procedures were as follows: 15 mg AceKGM was dissolved in 5 ml of organic solvent (acetone) at room temperature, and 2 mg of Cur was added to this organic solution away from light. Thereafter, the organic mixture solution was slowly added to 0.04% aqueous solution of TPGS under continuous stirring, using a syringe pump at a flow rate of 3 ml/min. The emulsion was stirred at room temperature away from light until the complete evaporation of organic solvents. Subsequently, excess Cur was removed by repeated centrifugation and washing with deionized water, until Cur could not be detected in the supernatant. Finally, the Cur–AceKGM NPs were dried and stored in a sterile container at 4°C for subsequent experiments.

### 2.3 Nanoparticle characterization

#### 2.3.1 Particle size and morphology characterization

The size distribution of the particles was measured by dynamic light scattering (DLS) (Zetasizer Nano ZS, Malvern Instruments, United Kingdom). Size measurement was performed in triplicate at room temperature. The morphology of the nanoparticles was observed by atomic force microscopy (AFM, Bruker, Germany). A drop of nanoparticles was spread on mica and imaged using Tap 150Al-G Silicon AFM probes in ScanAsyst mode. The nanoparticles were also observed using a scanning electron microscope (SEM, PHILIPS XL-30E, Netherlands) at an acceleration voltage of 10 kV. The samples were sputter-coated with gold using a sputtering device (Leica EM SCD005, Germany). All tests were performed in triplicate.

#### 2.3.2 Fourier transform infrared spectroscopy

Cur, AceKGM, and Cur–AceKGM NPs were analyzed by Fourier transform infrared (FT-IR) (Bruker Equinox 55, United States) spectroscopy using potassium bromide pellets. All spectra were obtained with an accumulation of 20 scans in the wave-number range of 4,000–500 cm^−1^ at a resolution of 4 cm^−1^.

#### 2.3.3 X-ray powder diffraction)

The crystal states of Cur, AceKGM, the physical mixture of Cur and AceKGM nanoparticles (Cur&NPs), and Cur–AceKGM NPs were studied using an automatic X-ray powder diffractometer (Miniflex600, Rigaku, Japan). The instrument was operated at 30 kW and 30 mA in the range (2θ) of 5–80°. The scan rate was 5°/min. The powder sample was equilibrated in a 100% RH chamber for 24 h at room temperature.

### 2.4 Drug encapsulation efficiency and *in vitro* drug release

A known mass of Cur–AceKGM NPs was dissolved in acetone. Subsequently, the solution was added drop-wise to 20 ml ethanol away from light, resulting in the precipitation of the AceKGM polymer matrix, while Cur remained soluble in ethanol. After centrifugation, the supernatant liquid was analyzed using a UV–vis spectrophotometer at 425 nm (Shimadzu, Japan). The drug content of Cur–AceKGM NPs was calculated from the standard curve. Drug entrapment efficiency (EE) was calculated using the following equation:

EE=(Mass of the drug in nanoparticles/Mass of drug added)×100%.

Cur release kinetics from Cur–AceKGM NPs was analyzed by soaking Cur–AceKGM NPs in SGF with pH 1.2 or simulated intestinal fluid (SIF) with pH 7.4. Both pH values were selected based on the normal variation of the gastrointestinal tract (GIT) in the stomach (pH ∼1.5) and the colon (pH 7∼7.8), respectively (Evans et al., 1988). Simulated gastric fluid was prepared by adding 0.1 N HCl, 2 g NaCl, and 3.5 g pepsase to 1 L deionized water. Simulated intestinal fluid was prepared by adding 102 mg KH_2_PO_4_ and 155 mg Na_2_HPO_4_·2H_2_O to 1 L SGF; subsequently, 0.01 M NaOH solution was used to adjust pH to 7.4, and then, 4 mg β-mannase was added (Burke et al., 2005). A sample (5 mg) of Cur–AceKGM NPs was weighed accurately and incubated in 20 ml SGF or SIF at 37°C with mild shaking. At pre-determined time intervals, 0.5 ml of the solution was collected and replaced with 0.5 ml of fresh medium to maintain the soaking condition. The solution was centrifuged at 15,000 rpm for 20 min at 4°C. The absorbance of the liquid supernatant was analyzed at 425 nm, and the size of the nanoparticles was measured DLS. All the experiments were performed in triplicate.

### 2.5 Activity of the released Cur

The activity of Cur released from nanoparticles was detected by measuring their capacity to eliminate the stable 1,1-diphenyl-2-picrylhydrazyl (DPPH) free radical, and the method was reported by Gharibi et al. (2015) with minor modification. The Cur–AceKGM NPs were soaked in SGF/SIF for 4 h. Then, 1 ml of the solution was centrifuged (15,000 r/min, 20 min, 4°C), and the sediment and DPPH (3 ml and 400 μM, respectively) in acetone were stirred and incubated in a dark place for 30 min. Wavelength scanning was carried out using a UV–vis spectrophotometer at 517 nm. DPPH degradation was calculated by the following equation:

$$DPPH\;scavenged\left(\%\right)=\frac{A0-Ai}{A0}\times 100\%,$$

where A_0_ is the absorption of the blank (DPPH + acetone), and A_i_ is the absorption of the sample (DPPH + acetone + Cur released from Cur–AceKGM NPs).

The Cur–AceKGM NPs were soaked in SGF/SIF for 4 h. Then, 1 ml of the solution was centrifuged (15,000 r/min, 20 min, 4°C), and the sediment was dissolved in DMSO and diluted by the complete medium. RAW264.7 cells were seeded in 24-well plates with a density of 5 × 10^4^ cells per well and incubated at 37°C for 24 h. Subsequently, the medium was replaced with a complete medium containing 1 μg/ml lipopolysaccharide (LPS) to stimulate the production of ROS, except for one group where replacement was with a complete medium without LPS. Then, one group of LPS-treated cells received no treatment, and the other groups were treated with the conditioned medium. After incubation of 24 h, the medium was removed and the cells were rinsed with serum-free medium three times; then, an ROS probe DCFDA (10 μM) was added and incubated in the dark for 30 min. The level of intracellular ROS was imaged using an inverted fluorescence microscope (excited at 488 nm).

### 2.6 *In vitro* biocompatibility test

#### 2.6.1 CCK-8 assay

To evaluate the cytotoxicity of Cur–AceKGM NPs, the sterile samples were dispersed in a culture medium. NIH3T3 or RAW264.7 cells were seeded at 5 × 10^4^ cells per well in 96-well plates, and then cultured in DMEM supplemented with 10% FBS for 24 h. Subsequently, the culture medium was replaced by the medium containing Cur–AceKGM NPs (100 μg/ml, 200 μg/ml, and 500 μg/ml, respectively). After incubation at 37°C for 24 h or 48 h, the CCK-8 assay was performed to check cell viability. All experiments were performed in triplicate. The percentage of relative cell viability was calculated using the following equation:

$$Cell\;viability\left(\%\right)=\frac{OD\;sample-OD\;control}{OD\;control}\times 100\%,$$

where OD is the designated optical density at 450 nm.

#### 2.6.2 Interaction of Cur–AceKGM NPs and cells

The RAW264.7 polarization protocols were carried out according to the following procedures: the M0 phenotype RAW264.7 cells were induced to M1 cells by the complete medium containing 1 μg/ml LPS and were induced to the M2 cells by the complete medium containing 20 ng/ml IL-4 (Wang et al., 2020). To evaluate the interaction between the Cur–AceKGM NPs and cells, NIH3T3 cells and various phenotypes of macrophage (M0, M1, and M2 phenotypes) cells were seeded at 1 × 10^4^ cells per well in 24-well plates. After incubation at 37°C for 24 h, 50 μg/ml Cur–AceKGM NP solution was added and incubated for 3 h, and the cells were washed three times with PBS. The various cells were fixed with 2.5% glutaraldehyde at room temperature for 10 min and then washed with PBS and imaged using an inverted fluorescence microscope.

### 2.7 *In vivo* test

Male Kunming mice (6–8 weeks, average weight approximately 20 g) were used for the *in vivo* test. The animal experiment was approved by the Animal Care and Experiment Committee of Jinan University. Mice were weighed and randomly divided into five groups: untreated group (control), physiological saline group, physical mixture of the Cur&NP group (Cur&NPs, dispersed in physiological saline), dexamethasone group (dissolved in physiological saline), and Cur–AceKGM NP group (*n* = 3). Acute DSS colitis was induced by the addition of 5% (w/w) DSS in the drinking water for the experiment groups and fresh DSS solutions were replaced daily. The control group received sterilized deionized water as drinking water. The colitis model was confirmed by the changes in mouse body weight, fecal humidity, and hematochezia.

On days 3, 4, 5, 6, and 7, the mice were intragastrically administered different test items as follows: physiological saline or Cur&NPs (50 mg Cur/kg body weight), dexamethasone (0.1 mg/kg body weight), or Cur–AceKGM NPs (50 mg Cur/kg body weight). Diarrhea, rectal bleeding, and the weight of the mice were observed during the experimental period. The disease activity index (DAI) was used to evaluate the grade of intestinal inflammation as previously described in Table 1(Wirtz et al., 2007). On day 8, the mice were sacrificed by excessive anesthesia, and colons were acquired for macroscopic observation, gauge length, MPO activity, ELISA, and H&E staining analysis. In addition, the histological score was executed according to a previous report (Ma et al., 2022). For the epithelium (E), the standards were as follows: 0, normal morphology; 1, loss of goblet cells; 2, loss of goblet cells in large areas; 3, loss of crypts; and 4, loss of crypts in large areas. Infiltration (I) was evaluated by the following standard scores: 0, no infiltrate; 1, infiltrate around the crypt basis; 2, infiltrate reaching the muscularis mucosae; 3, extensive infiltration reaching the muscularis mucosae and thickening of the mucosa with abundant edema; and 4, infiltration of the submucosa. The total histological score was presented as E + I.

**TABLE 1:** Standard of DAI scores.

Score	Weight loss	Stool consistency	Blood
0	<1%	Normal	Negative hemoccult
1	1–5	Soft but still formed	Positive hemoccult
2	6–10	Very soft	Blood traces in stool visible
3	>10%	Diarrhea	Rectal bleeding

### 2.8 Statistical analysis

All data were recorded as the mean ± standard deviation of three independent measurements unless specified otherwise. The data were analyzed with the statistical program Origin 8.5. Statistical analyses were carried out by conducting Student’s t-test and one-way analysis of variance (ANOVA). A *p*-value below 0.05 was considered significant.

## 3 Results

### 3.1 Nanoparticle preparation and characterization

**FIGURE 1:**
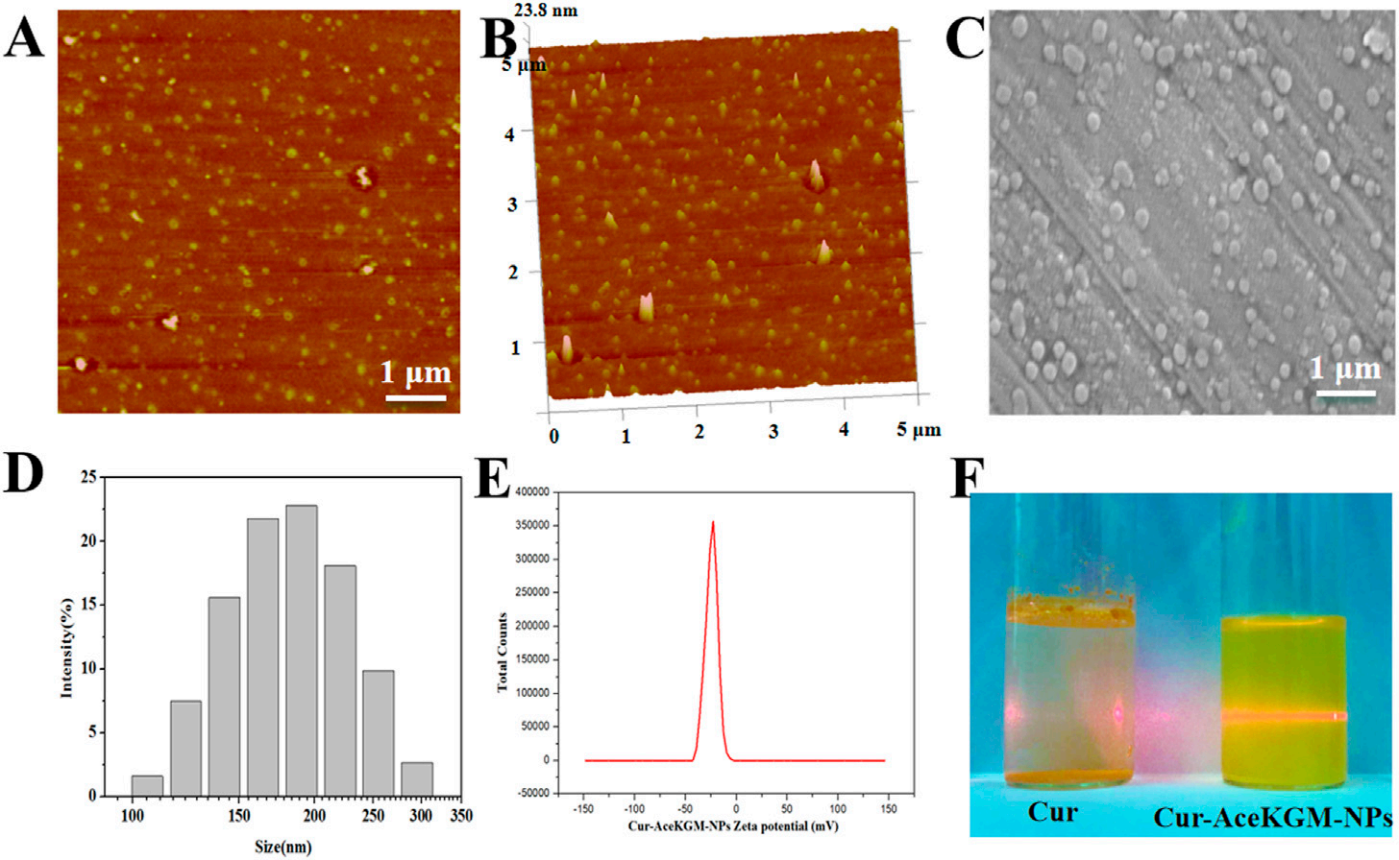
Atomic force microscopy images of Cur–AceKGM NPs. **(A)** 2D image and **(B)** 3D image. Scanning electron microscopy image of Cur–AceKGM-NPs **(C)**. Particle size distribution **(D)**and zeta potential **(E)** of Cur–AceKGM NPs. The dispersibility of Cur and Cur–AceKGM NPs in PBS **(F)**.

Cur–AceKGM NPs were prepared by emulsion solvent evaporation techniques. The morphology of the particles was observed by AFM (Figures 1A,B) and SEM (Figure 1C). The nanoparticles had a monodispersed size distribution with an average size of approximately 200 nm (the particles’ size distribution is shown in Figure 1D). The zeta potential of the nanoparticles was −24.3 ± 0.27 mV (Figure 1E). The EE and drug-loading efficiency were around 83 ± 3.5% and 8.5 ± 0.2 μg/mg, respectively. The dispersibility of Cur and Cur–AceKGM NPs in PBS is shown in Figure 1F. Cur was insoluble in the solution when aggregated; however, Cur–AceKGM NPs were completely dispersed in water and a clear yellow solution was observed.

### 3.2 FT-IR and XRD

**FIGURE 2:**
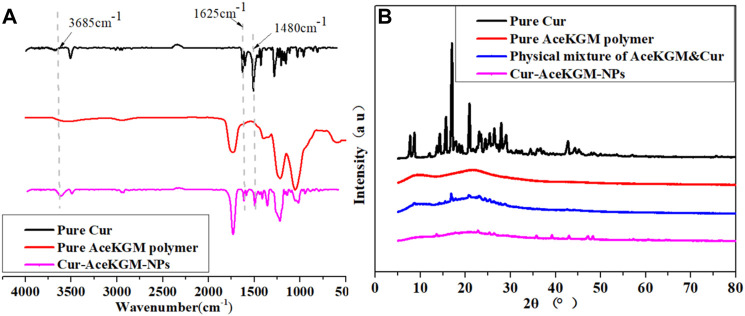
FT-IR **(A)** and XRD **(B)** spectra of pure Cur, pure AceKGM nanoparticles, physical mixture of AceKGM and Cur, and Cur–AceKGM NPs.

To further confirm the presence of Cur in nanoparticles, FT-IR analysis was conducted. The results (Figure 2A) showed that a band at 3,500 cm^−1^ was attributed to -OH group stretching vibration in Cur, and a strong peak at 1,480 cm^−1^ was observed in Cur and Cur–AceKGM NPs due to -CH_2_ bending vibration. The signature peaks at 1,625 cm^−1^ and 1,600 cm^−1^ were found in Cur and Cur–AceKGM NPs due to C=C double bonds and aromatic C=C double bonds, respectively. Furthermore, the absence of these peaks in AceKGM indicated that Cur was present in the nanoparticle.

The crystal state of Cur in NPs was reported to determine the drug release (Kumar et al., 2014). According to the XRD analysis results (Figure 2B), typical peaks of Cur were identified between 10 and 30°, which corresponds to its crystalline condition. On the contrary, the characteristic peaks of Cur were absent in Cur–AceKGM NPs. It meant that Cur was highly dispersed and did not aggregate into the crystal phase in the AceKGM nanoparticles.

### 3.3 *In vitro* drug release profile

**FIGURE 3:**
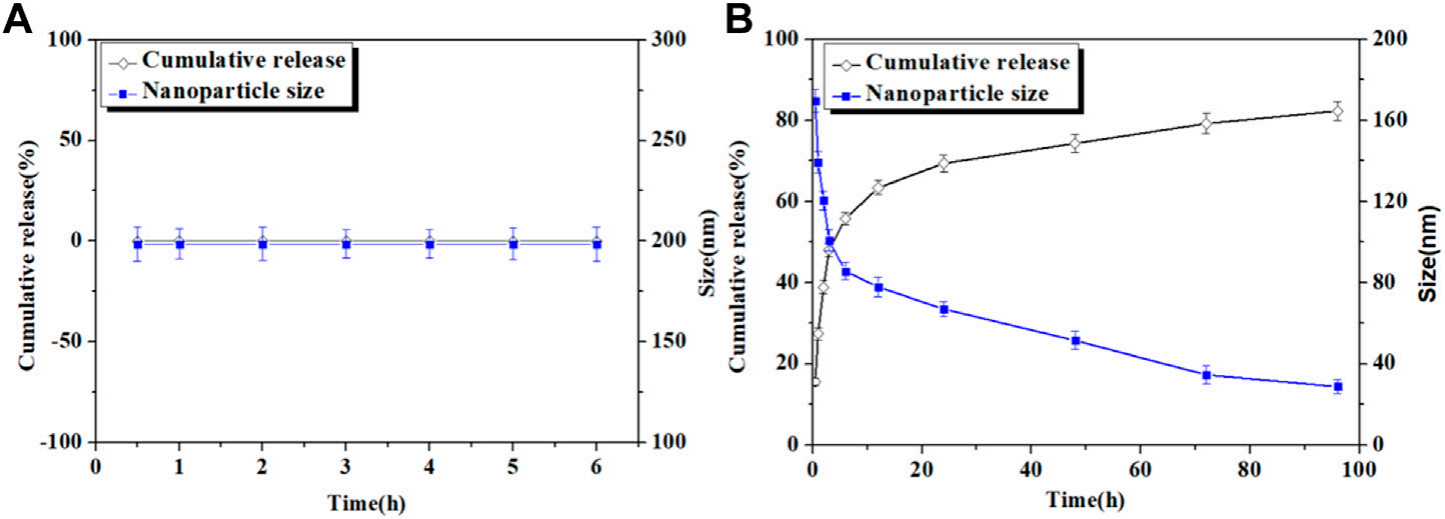
Nanoparticles’ size variation and *in vitro* release of Cur from Cur–AceKGM NPs in SGF **(A)** and SIF **(B)**; all the experiments were performed in triplicate.

The *in vitro* release behavior of Cur from Cur–AceKGM NPs was investigated in SGF or SIF. As shown in Figure 3A, the diameter of Cur–AceKGM NPs did not change significantly after 6 h of incubation in SGF, and only a little amount of Cur was released. In contrast, Cur–AceKGM NPs exhibited a sustained drug release of 81% in SIF in 48 h and their diameter gradually decreased to approximately 30 nm (Figure 3B). *In vitro* release results revealed that Cur from Cur–AceKGM NPs had no significant release in SGF but more than 60% release in SIF in 24 h, which indicates that Cur–AceKGM NPs might be an appropriate delivery vector for colon targeting.

### 3.4 Activity of the released Cur

**FIGURE 4:**
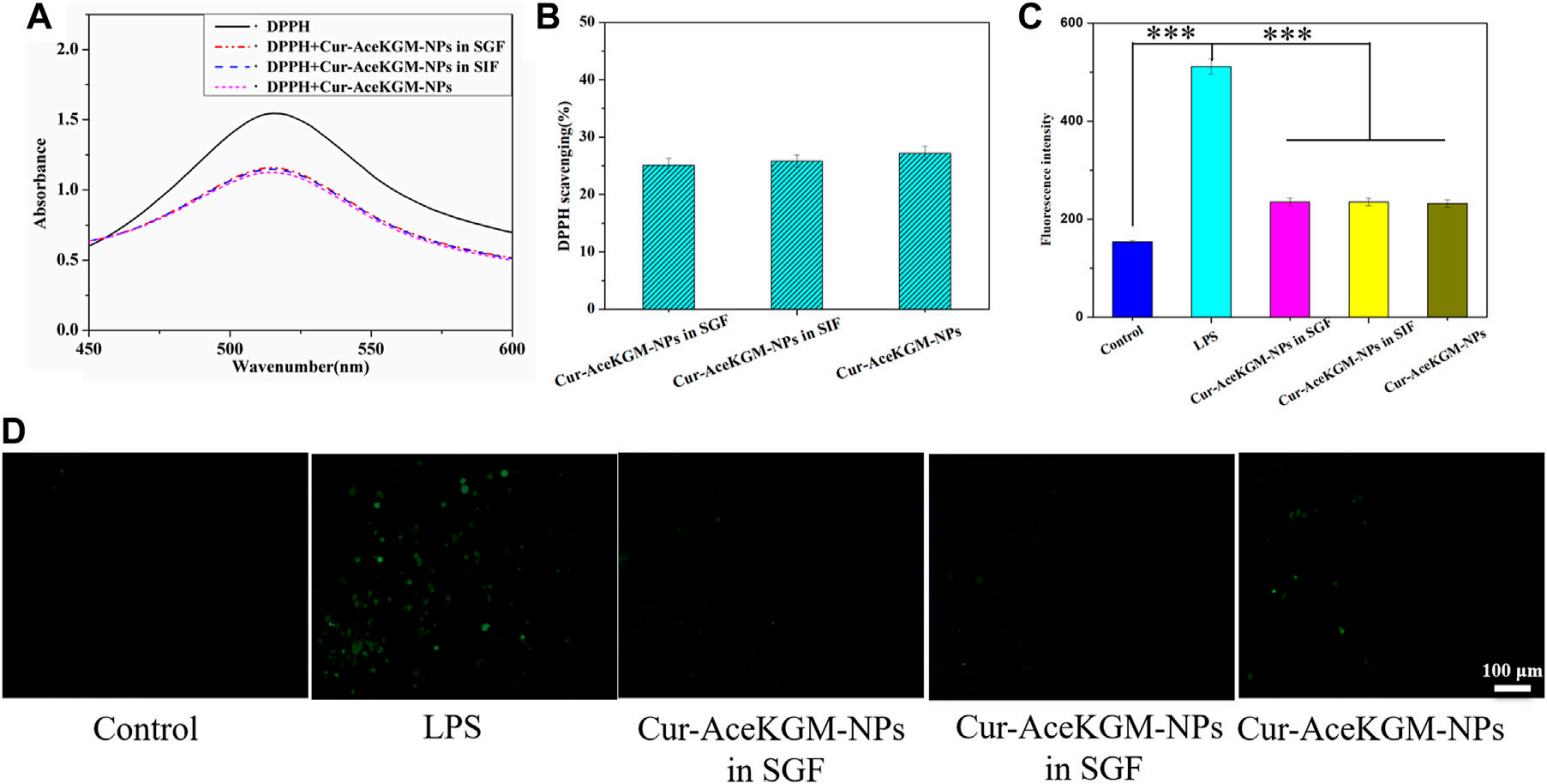
**(A)** UV−vis spectra of blank DPPH, DPPH + Cur–AceKGM NPs in SGF, DPPH + Cur–AceKGM NPs in SIF, and Cur–AceKGM NPs. **(B)** DPPH scavenging effect of the Cur–AceKGM NPs. **(C)** Fluorescence intensity of DCFDA in RAW264.7 cells with different treatments. **(D)** Fluorescence microscopy images of RAW264.7 cells with different treatments, green: ROS. All the experiments were performed in triplicate, scale bar = 100 μm, and data are shown as the mean ± SD. ***p < 0.001.

To examine the effect of processing conditions and the acidic environment in the stomach on the activity of the released Cur, the antioxidant activity of Cur released from Cur–AceKGM NPs in SGF was detected by measuring their capacity for scavenging the stable DPPH free radical. Figure 4A shows UV−vis wavelength scanning of DPPH, DPPH + nanoparticles incubated in SGF (DPPH + Cur–AceKGM NPs in SGF), DPPH + nanoparticles incubated in SIF (DPPH + Cur–AceKGM–NPs in SIF), and DPPH + Cur–AceKGM NPs. UV spectrum absorbance is proportional to the concentration of DPPH in the solution. The progressive decrease in the intensity of the DPPH peak indicated that the stable DPPH radical concentration was reduced after incubation with the nanoparticles. It suggested that the Cur loaded in the nanoparticles maintained the ability to scavenge the stable DPPH radicals in SGF or SIF. The DPPH scavenging results (Figure 4B) revealed no significant difference between untreated Cur–AceKGM NPs and Cur–AceKGM NPs in SGF or SIF (*p* > 0.05), and the activity of Cur in AceKGM nanoparticles was sustained after passing through the stomach and colon.

In addition to evaluating the antioxidant activities of Cur–AceKGM NPs in a cell-free environment, we further appraised their antioxidant capacities with macrophages. As shown in Figures 4C,D, the incubation of macrophages with LPS resulted in higher levels of ROS; nevertheless, incubation with Cur–AceKGM NPs inhibited the production of ROS by LPS.

### 3.5 Biocompatibility

**FIGURE 5:**
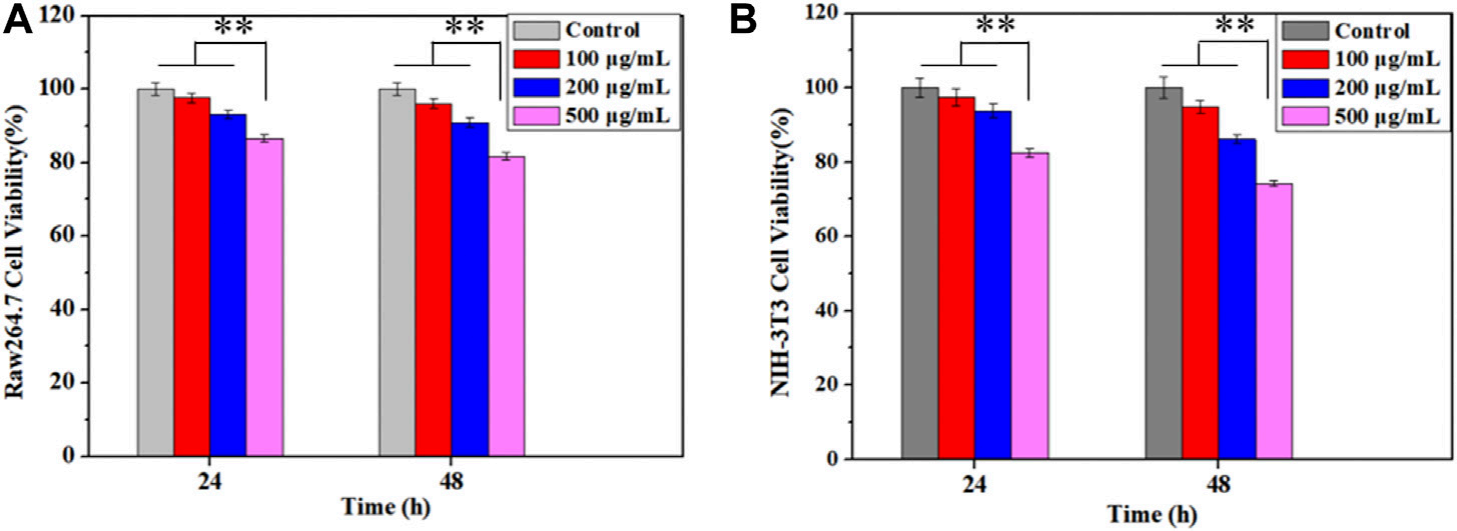
Effects of Cur–AceKGM NP concentrations on the proliferation of RAW264.7 **(A)** and NIH3T3 **(B)** cells cultured at 24 and 48 h; all the experiments were performed in triplicate and **p < 0.01.

The cytotoxicity of Cur–AceKGM NPs was examined with NIH3T3 cells and RAW264.7 cells by CCK-8 assay *in vitro*. The results (Figure 5) indicated that the cytotoxicity of nanoparticles was dose-dependent. As shown in Figure 5A, after culture with Cur–AceKGM NPs for 24 and 48 h, the survival of macrophages gradually decreased with the increase in nanoparticle concentration, and there was a similar trend to NIH3T3 cells. These findings indicated that Cur–AceKGM NPs exhibited low toxicity against both RAW264.7 cells and NIH3T3 cells at a high concentration (cell viability>80%) and could be used as a safe drug delivery system.

### 3.6 Interaction of Cur–AceKGM NPs and RAW264.7 or NIH3T3 cells

**FIGURE 6:**
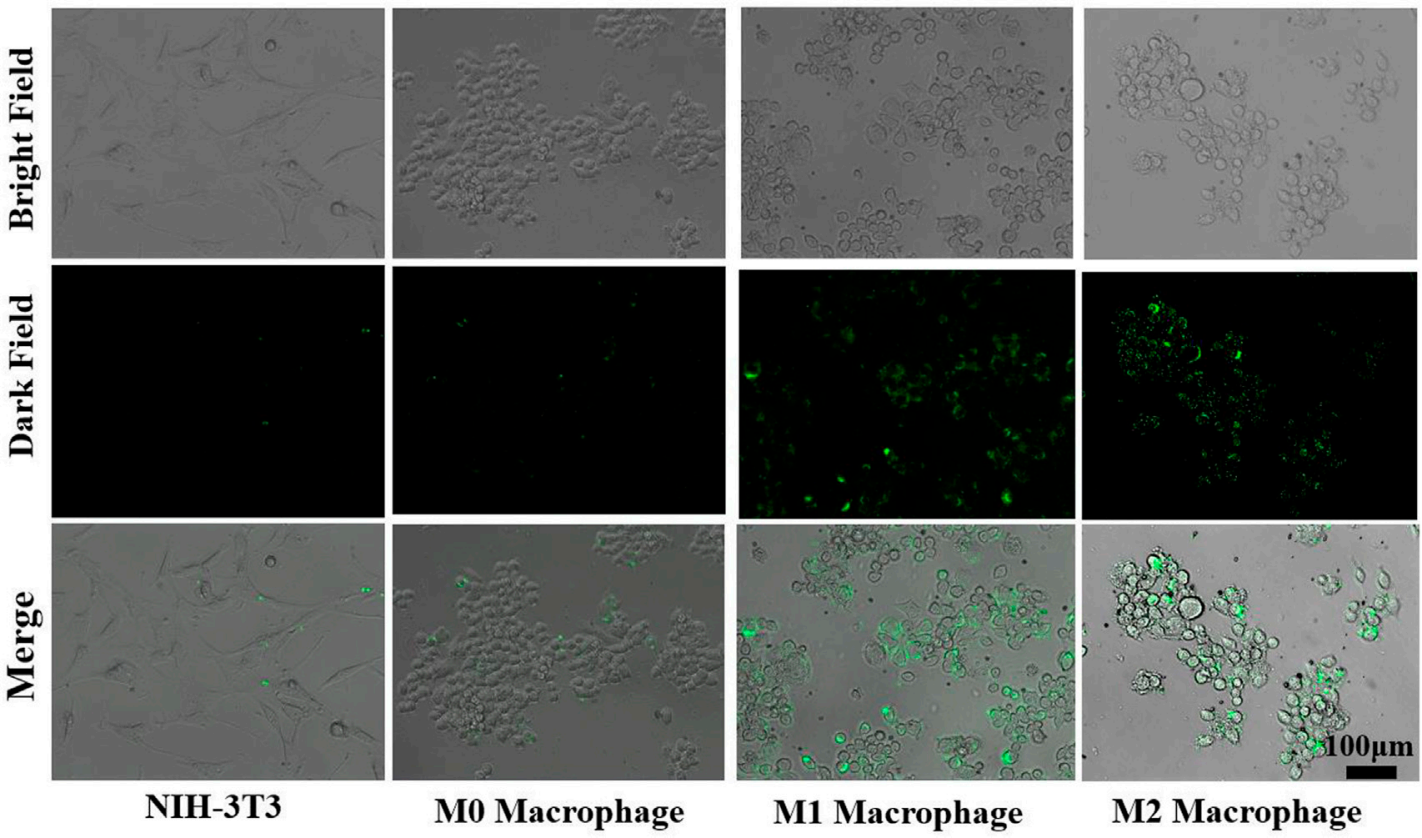
Adhesion of Cur–AceKGM NPs with NIH3T3 cell and RAW264.7 with different phenotypes; scale bar = 100 μm.

Duration of drugs in the colon plays a key role in improving the bioavailability of drugs and the healing of colon mucosa. Consequently, if Cur–AceKGM NPs attached to cells or tissues at the lesion site, they could continue to function. The photochemical characteristics of Cur were used through fluorescence microscopy to examine the extracellular adherence of Cur–AceKGM NPs with different cell types. Figure 6 shows the adhesion of Cur–AceKGM NPs on NIH3T3 cells and M0, M1, and M2 phenotype RAW264.7 cells. Low fluorescence could be observed on NIH3T3 cells and M0 phenotype RAW264.7 cells, indicating that there were few Cur–AceKGM–NPs on those cells. On the contrary, higher fluorescence intensity was found on M1 or M2 phenotype RAW264.7 cells treated with Cur–AceKGM NPs, which meant that Cur–AceKGM NPs could be “captured” more efficiently by both M1 and M2 phenotype RAW264.7 cells. These results revealed that Cur–AceKGM NPs tended to preferentially accumulate in inflamed tissue for avoiding rapid elimination by diarrhea and improving the bioavailability of Cur.

### 3.7 *In vivo* results

**FIGURE 7:**
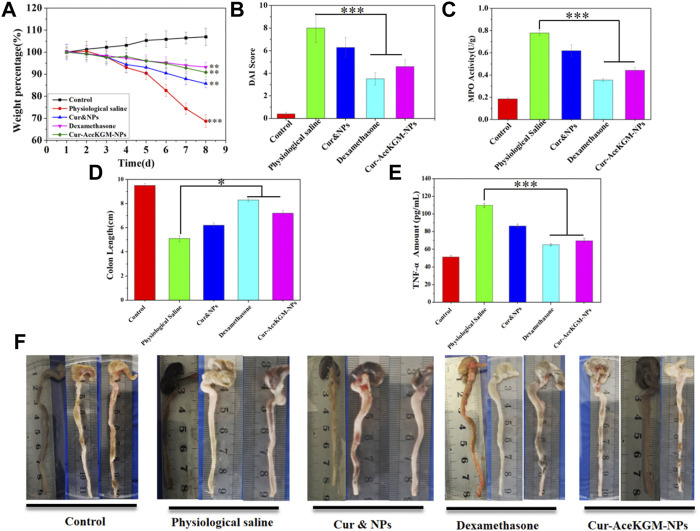
Therapeutic efficacy of Cur–AceKGM NPs in mice with DSS-induced colitis. **(A)** Body weight percentage variation during the treatment with physiological saline, physical mixture of curcumin and AceKGM nanoparticles (Cur&NPs), dexamethasone, and Cur–AceKGM NPs; **p < 0.01 and ***p < 0.001 *versus* the control group. **(B)** Disease activity index score. **(C)** MPO activity determination of the colon tissue. **(D)** Statistical analysis of colon length in different treatment groups, **(E)** colon tissue TNF-α amounts, measured by ELISA assays. **(F)** Gross view of the colon. All the experiments were performed in triplicate, and data are shown as mean ± SD. ***p < 0.001 and **p < 0.01.

Finally, the anti-inflammatory function of Cur–AceKGM NPs was investigated using a mouse colitis model. The DSS-induced colitis mice were treated with physiological saline, Cur&NPs, dexamethasone, and Cur–AceKGM NPs *via* intragastric administration once a day for 5 days, and the Cur–AceKGM NPs presented a favorable therapeutic efficacy. The mice subjected to 5% DSS solution feeding for 3 days developed severe colitis with bloody diarrhea, loss of appetite, and sustaining weight loss. As shown in Figure 7A, for the control group, body weight increased with feeding time but was significantly decreased in the IBD model groups. On the 8th day, in the physiological saline group, the mice showed a maximum loss of body weight (about 30%) and about 20% in the Cur&NP group, and presented a minimum weight loss (about 6%) in dexamethasone and Cur–AceKGM NPs. The increased DAI score and MPO activity were significantly suppressed by the treatment with dexamethasone and Cur–AceKGM NPs (Figures 7B,C). The gross examination of colons on the 8th day showed striking hyperemia, inflammation, and shorter colon length (Figure 7D). Mice treated with Cur–AceKGM NPs exhibited no significant macroscopic inflammation and longer colon length. Moreover, the Cur–AceKGM NPs effectively inhibited the release of inflammatory cytokines (Figure 7E). The gross view of the colon is shown in Figure 7F. All these results demonstrated a solid efficacy of Cur–AceKGM NPs in treating mice with DSS-induced IBD.

### 3.8 Histological analysis

**FIGURE 8:**
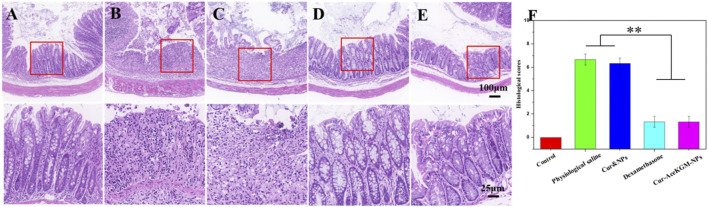
Histological analyses of colon sections stained with H&E from the control group **(A)**, physiological saline group **(B)**, physical mixture Cur and NP group **(C)**, dexamethasone group **(D)**, and Cur–AceKGM NP group **(E)**, respectively. **(F)** Corresponding histological scores. All the experiments were performed in triplicate; data are shown as mean ± SD. **p < 0.01.

Figure 8 shows the H&E staining results of the colon tissue treated with physiological saline, physical mixed Cur and NP group, dexamethasone, and Cur–AceKGM NPs. As shown in Figure 8, the mouse colon tissue of the mucosa and the submucosal muscle layer presented a normal, intact, and continuous structure in control groups. In the physiological saline group, both the mucosa and the muscle layer were seriously damaged, and there was submucosal edema. Meanwhile, the glandular cavity structure and cup cells almost disappeared, and a large number of inflammatory cells (mainly lymphocytes and necrotic neutrophils) infiltrated into the mucosal layer and penetrated the serosa. In the Cur&NP groups, the degree of inflammatory cell infiltration was not significantly different from that of the physiological saline group. For the dexamethasone and Cur–AceKGM NP treatment groups, only a small number of inflammatory cells could be observed in the mucosal surface, without significant inflammation in the mucosal, submucosal, and muscle layers. Furthermore, the structures of the mucosa and muscle layer in the Cur–AceKGM NP group were similar to those in the control group. The Cur–AceKGM NP group showed similar colonic histological appearances with the control group (Figures 8E,F). These results indicate that orally administered Cur–AceKGM NPs had a commendable therapeutic effect against colitis. All these results demonstrated that AceKGM nanoparticles could maintain Cur activity in the stomach and intestines and release drugs in the colon for treating DSS-induced colitis.

## 4 Discussion

The current study aimed to design a novel oral colon-targeted drug delivery system to accomplish the treatment requirements of colitis disease. Many other strategies such as a pH-sensitive delivery system, time-controlled one, and enzyme degradation one have shown promise in protecting drugs while passing through the stomach and intestinal tract and precisely releasing drugs in the colon (Xiao et al., 2015; Casati et al., 2020; Naeem et al., 2020). However, a strategy for desirable cell targeting and bioavailability with prolonged drug release is particularly challenging. Therefore, novel OCDDSs for efficient, specific drug delivery for IBD treatment is still a crucial gap for researchers to address. In the current study, AceKGM was used to create an oral colon-targeting drug delivery system to suit the needs of both sustained release in the colon and improved bioavailability.

A large number of studies have reported KGM or its derivatives as a colon-targeted delivery system (Chen et al., 2005; Ding et al., 2020). There were numerous reports of KGM or its derivatives on targeting macrophages (Huang et al., 2015; Gan et al., 2018). However, few reports on the dual-targeting delivery of both colon and colitis macrophages were available. In this study, KGM was modified by acetylation and anti-inflammatory Cur was loaded in AceKGM to meet the dual targeting requirement for the treatment of IBD. The acetylation of KGM was done for the following reasons: first, acetylation is an effective method to modify polysaccharides, to modulate their hydrophobicity and processability. Second, acetylation presented KGM an ideal matrix system for the incorporation of hydrophobic drugs, such as Cur. In addition, acetylation is a common processing mode in cells, which endows biomacromolecules with new biological activity. Our previous study confirmed that acetylation is effective in promoting the macrophage targeting of KGM. In the present study, more Cur–AceKGM NPs aggregated on the surface of macrophages than of NIH3T3 cells. This phenomenon was attributed to the higher selective adhesive force between AceKGM and macrophages than that of NIH3T3 cells (Wang et al., 2020). Moreover, the mean particle size of the Cur–AceKGM NPs was found to be about 200 nm, which was confirmed by DLS, AFM, and SEM. It has been reported that nanoparticles with a size of 100–200 nm could penetrate through submucosal layers and flourish at the site of colon inflammation (Lamprecht et al., 2001; Ali et al., 2014). Hence, acetylation and nano-size could endow AceKGM NPs with steady adhesion in the colon, which would be beneficial to avoid rapid elimination by diarrhea and improve the bioavailability of Cur.

Controlled release is essential for enhancing medication absorption (He et al., 2021; Tang et al., 2021; Ren et al., 2022). In previous reports, KGM was designed as a delivery vehicle because of its swelling and biodegradable characteristic, and it also demonstrated excellent therapeutic efficacy against IBD. However, one major concern of this type of delivery vehicle is the swelling or degradation of the delivery vehicle and premature drug release in the stomach or intestine, which not only leads to decreased drug delivery to the colon but also often generates various side effects after drug absorption in the stomach or intestine. In the present work, we observed that the diameter of nanoparticles hardly changes after being treated with SGF. Simultaneously, only a trace amount of Cur was detected. It indicated that AceKGM did not swell or degrade significantly in SGF. We speculate that the acetylation of KGM prevents the acidic buffer from permeating into the AceKGM microsphere. On the contrary, the diameter of the Cur–AceKGM NPs decreased and a large amount of Cur was detected after being incubated in SIF containing β-mannanase because AceKGM may have been degraded by β-mannanase, resulting in a burst release of the loaded Cur. It implied that AceKGM still maintained similar β-mannanase susceptibility to KGM, despite the rate of degradation being reduced. As reported and successfully conducted in a previous study (Huang et al., 2015), cationic konjac glucomannan could be a promising and reliable delivery material for IBD treatment. Moreover, the amorphous state of Cur was confirmed by XRD. The dispersed state of Cur would benefit the continuous release of Cur from NPs and improve the bioavailability of Cur. These results are in agreement with the previous reports (Kumar et al., 2014).

The stable DPPH free radical and ROS scavenging results demonstrated there was no significant difference between the scavenging capacity of Cur–AceKGM NPs with and without digestive fluid. It indicated that the Cur–AceKGM NP delivery vehicle could retain the integrity and function of Cur. It is mainly AceKGM which may prevent the acidic buffer from completely permeating into the microsphere, maintaining the bioactivity of Cur. The *in vivo* experiments proved that Cur–AceKGM NPs decreased the MPO activity and promoted mice survival by scavenging oxygen radicals, reducing inflammatory cytokine production, and promoting colonic mucosal regeneration. These results are in agreement with the previous reports (Chen et al., 2009). The reasons were that AceKGM nanoparticles could deliver Cur into the colon lesion site and retain the bioactivity of Cur. Notably, the retention time of Cur–AceKGM NPs was extended by the specific adhesion of macrophages at the colon inflammation site, combined with a sustained release of Cur, which resulted in decreasing inflammatory response and exhibited significantly improved therapeutic efficacy against colitis in mice.

IBD is one of the most remarkable medical challenges worldwide. There is no ideal therapeutic treatment in the clinic. In the present study, we found that Cur–AceKGM NPs dually targeting the colon and colonic macrophages could be used as an effective delivery vehicle in the treatment of IBD. First, oral Cur–AceKGM NPs have better patient compliance than many other drug formulations such as rectal gel, enemas, and suppositories delivered through the rectum. Second, Cur–AceKGM NPs were relatively stable in the stomach and intestines with little premature drug release. Third, the loaded Cur was mainly released in a specific, controlled manner in the colon, acquiring an improved bioavailability and prolonged residence time of Cur in the colon, resulting in improved therapeutic efficacy against IBD in the clinic.

## 5 Conclusion

In conclusion, we developed an orally administered colon and macrophage-targeted drug delivery vehicle based on AceKGM and AceKGM-adhered cells by macrophage-specific monosaccharide residue receptors, demonstrating anti-inflammation activity in an experimental colitis model. AceKGM nanoparticles could protect the bioactivity of Cur while traversing the stomach and intestines, and release drugs exactly at the colon location. Simultaneously, Cur–AceKGM NPs accumulated on the inflammatory macrophage *via* high selective adhesion, effectively prolonging the release of Cur in the colon and enhancing the bioavailability of Cur. The oral colon-targeting drug delivery system showed anti-inflammatory activity and supported the therapeutic efficacy against IBD. Hence, this novel oral Cur delivery vehicle targeting colonic macrophages suggests a possible therapeutic method for the treatment of IBD.

## Data Availability

The original contributions presented in the study are included in the article/supplementary material; further inquiries can be directed to the corresponding author.

## References

[B1] Abraham C. Cho J. H. (2009). Inflammatory bowel disease. N. Engl. J. Med. Overseas. Ed. 361, 2066–2078. 10.1056/nejmra0804647 PMC3491806 19923578

[B2] Ali H. Weigmann B. Neurath M. F. Collnot E. M. Windbergs M. Lehr C. M. (2014). Budesonide loaded nanoparticles with pH-sensitive coating for improved mucosal targeting in mouse models of inflammatory bowel diseases. J. Control. Release 183, 167–177. 10.1016/j.jconrel.2014.03.039 24685705

[B3] Arya P. Pathak K. (2014). Assessing the viability of microsponges as gastro retentive drug delivery system of curcumin: Optimization and pharmacokinetics. Int. J. Pharm. X. 460 (1-2), 1–12. 10.1016/j.ijpharm.2013.10.045 24184218

[B4] Baliga M. S. Joseph N. Venkataranganna M. V. Saxena A. Ponemone V. Fayad R. (2012). Curcumin, an active component of turmeric in the prevention and treatment of ulcerative colitis: Preclinical and clinical observations. Food Funct. 3 (11), 1109. 10.1039/c2fo30097d 22833299

[B5] Behera S. S. Ray R. C. (2016). Konjac glucomannan, a promising polysaccharide of Amorphophallus konjac K. Koch in health care. Int. J. Biol. Macromol. 92, 942–956. 10.1016/j.ijbiomac.2016.07.098 27481345

[B6] Burke M. D. Park J. O. Srinivasarao M. Khan S. A. (2005). A novel enzymatic technique for limiting drug mobility in a hydrogel matrix. J. Control. Release 104 (1), 141–153. 10.1016/j.jconrel.2005.01.017 15866341

[B7] Casati F. Melocchi A. Moutaharrik S. Uboldi M. Foppoli A. Maroni A. (2020). Injection molded capsules for colon delivery combining time-controlled and enzyme-triggered approaches. Int. J. Mol. Sci. 21 (6), 1917. 10.3390/ijms21061917 32168895 PMC7139580

[B8] Chen C. Johnston T. D. Jeon H. Gedaly R. McHugh P. R. Burke T. G. (2009). An *in vitro* study of liposomal curcumin: Stability, toxicity and biological activity in human lymphocytes and Epstein-Barr virus-transformed human B-cells. Int. J. Pharm. X. 366 (1-2), 133–139. 10.1016/j.ijpharm.2008.09.009 18840516

[B9] Chen L.-G. Liu Z.-L. Zhuo R.-X. (2005). Synthesis and properties of degradable hydrogels of konjac glucomannan grafted acrylic acid for colon-specific drug delivery. Polymer 46 (16), 6274–6281. 10.1016/j.polymer.2005.05.041

[B10] Ding Y. F. Sun T. Li S. Huang Q. Yue L. Zhu L. (2020). Oral colon-targeted konjac glucomannan hydrogel constructed through noncovalent cross-linking by cucurbit[8]uril for ulcerative colitis therapy. ACS Appl. Bio Mat. 3 (1), 10–19. 10.1021/acsabm.9b00676 35019421

[B11] Evans D. F. Pye G. Bramley R. Clark A. G. Dyson T. J. Hardcastle J. D. (1988). Measurement of gastrointestinal pH profiles in normal ambulant human subjects. Gut 29 (8), 1035–1041. 10.1136/gut.29.8.1035 3410329 PMC1433896

[B12] Gan J. Dou Y. Li Y. Wang Z. Wang L. Liu S. (2018). Producing anti-inflammatory macrophages by nanoparticle-triggered clustering of mannose receptors. Biomaterials 178, 95–108. 10.1016/j.biomaterials.2018.06.015 29920405

[B13] Gharibi R. Yeganeh H. Rezapour-Lactoee A. Hassan Z. M. (2015). Stimulation of wound healing by electroactive, antibacterial, and antioxidant polyurethane/siloxane dressing membranes: *In vitro* and *in vivo* evaluations. ACS Appl. Mat. Interfaces 7 (43), 24296–24311. 10.1021/acsami.5b08376 26473663

[B14] Gupta S. C. Patchva S. Aggarwal B. B. (2013). Therapeutic roles of curcumin: Lessons learned from clinical trials. AAPS J. 15 (1), 195–218. 10.1208/s12248-012-9432-8 23143785 PMC3535097

[B15] He M. Qin Z. Liang X. He X. Zhu B. Lu Z. (2021). A pH-responsive mesoporous silica nanoparticles-based drug delivery system with controlled release of andrographolide for OA treatment. Regen. Biomater. 8 (4), rbab020. 10.1093/rb/rbab020 34221446 PMC8242227

[B16] Hu Q. Luo Y. (2021). Chitosan-based nanocarriers for encapsulation and delivery of curcumin: A review. Int. J. Biol. Macromol. 179, 125–135. 10.1016/j.ijbiomac.2021.02.216 33667554

[B17] Huang Z. Gan J. Jia L. Guo G. Wang C. Zang Y. (2015). An orally administrated nucleotide-delivery vehicle targeting colonic macrophages for the treatment of inflammatory bowel disease. Biomaterials 48, 26–36. 10.1016/j.biomaterials.2015.01.013 25701029

[B18] Kumar S. S. Mahesh A. Mahadevan S. Mandal A. B. (2014). Synthesis and characterization of curcumin loaded polymer/lipid based nanoparticles and evaluation of their antitumor effects on MCF-7 cells. Biochimica Biophysica Acta - General Subj. 1840 (6), 1913–1922. 10.1016/j.bbagen.2014.01.016 24440669

[B19] Lamprecht A. (2010). Ibd: Selective nanoparticle adhesion can enhance colitis therapy. Nat. Rev. Gastroenterol. Hepatol. 7 (6), 311–312. 10.1038/nrgastro.2010.66 20523352

[B20] Lamprecht A. Scha¨fer U. Lehr C.-M. Schafer U. (2001). Size-dependent bioadhesion of micro- and nanoparticulate carriers to the inflamed colonic mucosa. Pharm. Res. 18 (6), 788–793. 10.1023/a:1011032328064 11474782

[B21] Louiselle A. E. Niemiec S. Dewberry L. K. Stager M. Azeltine M. Krebs M. D. (2020). Local cerium oxide nanoparticle-miR146a delivery using chitosan gel decreases tumor necrosis factor-alpha expression in inflammatory bowel disease. J. Am. Coll. Surg. 231 (4), S205–S206. 10.1016/j.jamcollsurg.2020.07.760

[B22] Lu L. Chen G. Qiu Y. Li M. Liu D. Hu D. (2016). Nanoparticle-based oral delivery systems for colon targeting: Principles and design strategies. Sci. Bull. 61 (9), 670–681. 10.1007/s11434-016-1056-4

[B23] Ma Y. Duan L. Sun J. Gou S. Chen F. Liang Y. (2022). Oral nanotherapeutics based on *Antheraea pernyi* silk fibroin for synergistic treatment of ulcerative colitis. Biomaterials 282, 121410. 10.1016/j.biomaterials.2022.121410 35202934

[B24] Naeem M. Awan U. A. Subhan F. Cao J. Hlaing S. P. Lee J. (2020). Advances in colon-targeted nano-drug delivery systems: Challenges and solutions. Arch. Pharm. Res. 43 (1), 153–169. 10.1007/s12272-020-01219-0 31989477

[B25] Ng S. C. Shi H. Y. Hamidi N. Underwood F. E. Tang W. Benchimol E. I. (2017). The worldwide incidence and prevalence of inflammatory bowel disease in the 21 st century: A systematic review of population-based studies. Gastroenterology 152 (5), S970–S971. 10.1016/s0016-5085(17)33292-4 29050646

[B26] Ren X. Hu Y. Chang L. Xu S. Mei X. Chen Z. (2022). Electrospinning of antibacterial and anti-inflammatory Ag@hesperidin core-shell nanoparticles into nanofibers used for promoting infected wound healing. Regen. Biomater. 9, rbac012. 10.1093/rb/rbac012 35592139 PMC9113224

[B27] Sethi S. SaruchiKaith B. S. Kaur M. Sharma N. Kumar V. (2020). Cross-linked xanthan gum–starch hydrogels as promising materials for controlled drug delivery. Cellulose 27 (8), 4565–4589. 10.1007/s10570-020-03082-0

[B28] Song W. B. Wang Y. Y. Meng F. S. Zhang Q. H. Zeng J. Y. Xiao L. P. (2010). Curcumin protects intestinal mucosal barrier function of rat enteritis via activation of MKP-1 and attenuation of p38 and NF-κB activation. PLoS One 5 (9), e12969. 10.1371/journal.pone.0012969 20885979 PMC2945766

[B29] Tang D. Wang Y. Wijaya A. Liu B. Maruf A. Wang J. (2021). ROS-responsive biomimetic nanoparticles for potential application in targeted anti-atherosclerosis. Regen. Biomater. 8 (4), rbab033. 10.1093/rb/rbab033 34285811 PMC8286794

[B30] Tang J. Chen J. Guo J. Wei Q. Fan H. (2018). Construction and evaluation of fibrillar composite hydrogel of collagen/konjac glucomannan for potential biomedical applications. Regen. Biomater. 5 (4), 239–250. 10.1093/rb/rby018 30094063 PMC6077832

[B31] Wang C. Li B. Chen T. Mei N. Wang X. Tang S. (2020). Preparation and bioactivity of acetylated konjac glucomannan fibrous membrane and its application for wound dressing. Carbohydr. Polym. 229, 115404. 10.1016/j.carbpol.2019.115404 31826490

[B32] Wirtz S. Neufert C. Weigmann B. Neurath M. F. (2007). Chemically induced mouse models of intestinal inflammation. Nat. Protoc. 2 (3), 541–546. 10.1038/nprot.2007.41 17406617

[B33] Xiao B. Si X. Zhang M. Merlin D. (2015). Oral administration of pH-sensitive curcumin-loaded microparticles for ulcerative colitis therapy. Colloids Surfaces B Biointerfaces 135, 379–385. 10.1016/j.colsurfb.2015.07.081 26275840 PMC4695233

[B34] Xu Q. Huang W. Jiang L. Lei Z. Li X. Deng H. (2013). KGM and PMAA based pH-sensitive interpenetrating polymer network hydrogel for controlled drug release. Carbohydr. Polym. 97 (2), 565–570. 10.1016/j.carbpol.2013.05.007 23911486

[B35] Zhang C. Chen J. D. Yang F. Q. (2014). Konjac glucomannan, a promising polysaccharide for OCDDS. Carbohydr. Polym. 104, 175–181. 10.1016/j.carbpol.2013.12.081 24607175

[B36] Zhang M. Merlin D. (2018). Nanoparticle-based oral drug delivery systems targeting the colon for treatment of ulcerative colitis. Inflamm. Bowel Dis. 24 (7), 1401–1415. 10.1093/ibd/izy123 29788186 PMC6085987

[B37] Zhao P. Xia X. Xu X. Leung K. K. C. Rai A. Deng Y. (2021). Nanoparticle-assembled bioadhesive coacervate coating with prolonged gastrointestinal retention for inflammatory bowel disease therapy. Nat. Commun. 12 (1), 7162. 10.1038/s41467-021-27463-6 34887414 PMC8660811

